# In Vitro and In Vivo Cell Uptake of a Cell-Penetrating Peptide Conjugated with Fluorescent Dyes Having Different Chemical Properties

**DOI:** 10.3390/cancers13092245

**Published:** 2021-05-07

**Authors:** Hideo Takakura, Honoka Sato, Kohei Nakajima, Motofumi Suzuki, Mikako Ogawa

**Affiliations:** Laboratory of Bioanalysis and Molecular Imaging, Graduate School of Pharmaceutical Sciences, Hokkaido University, Sapporo 060-0812, Hokkaido, Japan; htakakura@pharm.hokudai.ac.jp (H.T.); h_sato_2134@frontier.hokudai.ac.jp (H.S.); knakajima@pharm.hokudai.ac.jp (K.N.); suzukimo@hirakata.kmu.ac.jp (M.S.)

**Keywords:** peptide, fluorescence imaging, charge, hydrophilicity/lipophilicity

## Abstract

**Simple Summary:**

In fluorescence imaging employing a targeting strategy, fluorescent dyes conjugated with ligands may alter the pharmacokinetics of the conjugates. The aim of this study was to investigate whether in vitro and in vivo cell uptake are affected when fluorescent dyes with different chemical properties are conjugated with a ligand. The results show that attention should be paid to the chemical properties of fluorescent dyes in designing fluorescent imaging agents.

**Abstract:**

In molecular imaging, a targeting strategy with ligands is widely used because specificity can be significantly improved. In fluorescence imaging based on a targeting strategy, the fluorescent dyes conjugated with ligands may affect the targeting efficiency depending on the chemical properties. Herein, we used a cell-penetrating peptide (CPP) as a ligand with a variety of fluorescent cyanine dye. We investigated in vitro and in vivo cell uptake of the dye-CPP conjugates when cyanine dyes with differing charge and hydrophilicity/lipophilicity were used. The results showed that the conjugates with positively charged and lipophilic cyanine dyes accumulated in cancer cells in vitro, but there was almost no accumulation in tumors in vivo. On the other hand, the conjugates with negatively charged and hydrophilic cyanine dyes did not accumulate in cancer cells in vitro, but fluorescence was observed in tumors in vivo. These results show that there are some cases in which the cell uptake of the dye-peptide conjugates may differ significantly between in vitro and in vivo experiments due to the chemical properties of the fluorescent dyes. This suggests that attention should be paid to the chemical properties of fluorescent dyes in fluorescence imaging based on a targeting strategy.

## 1. Introduction

A targeting strategy with compounds that bind to specific biomolecules, so-called ligands, is widely used for molecular imaging and drug delivery. Due to ligands selectively binding to target cells, specificity can be significantly improved compared with a passive accumulation strategy [1,2]. Among various ligands, including antibodies, macromolecules, and small molecules, peptides are useful due to their tunable targeting properties (i.e., multi-valent peptides) and facile synthesis at a relatively low cost [3,4,5]. Among peptides suitable for a targeting strategy, cell-penetrating peptides (CPPs) are attractive candidates since they can be used as carriers for molecular imaging and as drug delivery vehicles for therapeutic compounds that do not easily cross the cellular membrane [6,7,8]. Usually, CPPs consist of short positively charged peptides with 5–30 amino acids, such as TAT and R8. Recently, CPPs specific to the target cells have been developed [9,10]. Peptides are becoming increasingly used as targeting molecules.

Fluorescence imaging is a useful method that allows for repeatable measuring in a non-invasive manner. It has been utilized in research and clinical practice to evaluate drug delivery and diagnose diseases. In fluorescence imaging based on a targeting strategy, fluorescent dyes conjugated with the ligands may affect the binding of the ligands to the target molecules and the pharmacokinetics of the conjugates. This is because fluorescent dyes are usually highly lipophilic due to their large π-conjugated systems; they also have charged functional groups to improve their solubility in aqueous solutions, thus changing the overall lipophilicity, hydrophilicity, and net charge of the conjugates. Therefore, the targeting efficiency of conjugates with large molecules, such as antibodies, as well as ligands of low molecular weight are affected by the chemical properties of the fluorescent dyes [11,12,13,14,15,16].

Herein, we investigated the influence of fluorescent dyes on cell uptake with CPP as the ligand and a variety of cyanine dyes as the fluorescent dye. As a model CPP, we used peptide A, which was discovered by Kondo et al. Cy3/Cy5 were chosen since they are often used as labeling dyes due to their good optical properties, including a high molar extinction coefficient and relatively high fluorescent quantum yield, and availability. For the investigation, we used cyanine dyes with different chemical properties, such as charge and hydrophilicity/lipophilicity (i.e., Cy3/Cy5 and sulfonated Cy3 (sulfoCy3)/Cy5 (sulfoCy5)). We compared in vitro and in vivo cell uptake of the dye-CPP conjugates.

## 2. Materials and Methods

### 2.1. Reagents and General Information

General chemicals were of the best grade available, supplied by FUJIFILM Wako Pure Chemical Corporation (Osaka, Japan), Tokyo Chemical Industries Co., Ltd. (Tokyo, Japan), KANTO CHEMICAL Co., INC. (Tokyo, Japan), and Sigma-Aldrich Co. LLC (St. Louis, MO, USA), and were used without further purification. Cy3/Cy5-SE was purchased from Abcam (Cambridge, UK) and sulfoCy3/sulfoCy5-SE was purchased from AAT Bioquest (Sunnyvale, CA, USA). Peptide A was purchased from GlyTech, Inc. (Kyoto, Japan). MALDI-TOF mass spectra (MS) were measured with an Autoflex III TOF/TOF (Bruker Corporation, Billerica, MA, USA). Purification and high-performance liquid chromatography (HPLC) analyses were performed by HPLC systems (Shimadzu Corporation, Kyoto, Japan) with reverse-phase columns: the COSMOSIL 5C_18_-AR-300 (4.6 × 150 mm) (NACALAI TESQUE, INC., Kyoto, Japan) for analysis and the COSMOSIL 5C_18_-AR-300 (10 mm × 150 mm) (NACALAI TESQUE, INC.) for purification, using eluent A (H_2_O/0.1% trifluoroacetic acid (TFA)) and eluent B (CH_3_CN/0.1% TFA). Fluorescence images of living cells were taken on a BX41 upright fluorescent microscope (Olympus Corporation, Tokyo, Japan) equipped with a suitable filter set for Cy3/Cy5 dyes; it consisted of a 530–550 nm excitation filter and a 575–625 nm emission filter for the Cy3 dye, and a 608–648 nm excitation filter and a 652–732 nm emission filter for the Cy5 dye. Flowcytometry was performed with a Gallios flow cytometer (Beckman Coulter, Inc., Brea, CA, USA). Fluorescence images of animals and organs were taken with a FluorVivo Imaging System (Indec BioSystems, Santa Clara, CA, USA) equipped with a suitable filter set for a Cy5 dye; it consisted of a 600–640 nm excitation filter and a 665 nm long pass filter. All experiments were carried out at room temperature, unless otherwise specified. 

### 2.2. Synthesis

Cy5-peptide A. Peptide A (0.74 mg) and Cy5 succinimidyl ester (SE) (0.20 mg, 0.32 μmol) were dissolved in 0.1 mL of DMSO, and 0.1 mL of 1 mM Na_2_HPO_4_aq was added to the solution. The reaction mixture was stirred for 1 h at 50 °C. Then, 0.2 mL of TFA was added to the reaction mixture. The mixture was further stirred for 30 min at 50 °C. The product was purified by semi-preparative reverse-phase HPLC (eluent A: H_2_O/1% TFA, eluent B: 99% CH_3_CN/0.1% TFA, A:B = 70:30 to 0:100 in 20 min) to produce a blue solid (0.41 mg). The HPLC chart of Cy5-peptide A is shown in Figure S1.

sulfoCy5-peptide A. sulfoCy5-peptide A was synthesized from peptide A and sulfoCy5-SE by the same method as that used to obtain Cy5-peptide A. The product was purified by semi-preparative reverse-phase HPLC (A:B = 80:20 to 20:80 in 25 min) to produce a blue solid. The HPLC chart of sulfoCy5-peptide A is shown in Figure S1.

Cy3-peptide A. Cy3-peptide A was synthesized from peptide A and Cy3-SE by the same method as that used to obtain Cy5-peptide A. The product was purified by semi-preparative reverse-phase HPLC (A:B = 70:30 to 0:100 in 20 min) to produce a red solid. The HPLC chart of Cy3-peptide A is shown in Figure S1.

sulfoCy3-peptide A. sulfoCy3-peptide A was synthesized from peptide A and sulfoCy3-SE by the same method as that used to obtain Cy5-peptide A. The product was purified by semi-preparative reverse-phase HPLC (A:B = 80:20 to 20:80 in 25 min) to produce a red solid. The HPLC chart of sulfoCy3-peptide A is shown in Figure S1.

### 2.3. Cell Culture

BxPC3 cells were gifted from Dr. Eisaku Kondo (Niigata University). Normal human dermal fibroblasts (NHDF) cells were purchased from Lonza (Basel, Switzerland). BxPc3 and NHDF cells were cultured in Dulbecco’s modified Eagle’s medium (DMEM; high glucose) (Sigma-Aldrich) and supplemented with 10% fetal bovine serum (FBS) (Thermo Fisher Scientific, Waltham, MA, USA) and 1% penicillin/streptomycin (NACALAI TESQUE, INC.). Cells were cultured at 37 °C in a CO_2_/air (5%/95%) incubator.

### 2.4. Fluorescence Microscopy

BxPC3 and NHDF cells were incubated on 35 mm culture dishes with a cover slip in DMEM with 10% FBS at 37 °C in 5% CO_2_. We added 0.1 μM (final) dye-peptide A analogues to the dishes and the cells were incubated for 3 or 24 h. Then, after washing with PBS, the cover slip was placed on a slide glass. For colocalization with lysosome, 50 nM (final) LysoTracker Green (Thermo Fisher Scientific) was added to the dishes 2 h before observation. Fluorescence images of live cells were captured using an upright fluorescence microscope with a 40× objective lens. The obtained images were analyzed using ImageJ software ver. 1.51.

### 2.5. Flow Cytometry

BxPC3 and NHDF cells were incubated on 35 mm culture dishes in DMEM with 10% FBS at 37 °C in 5% CO_2_. We added 0.1 μM (final) dye-peptide A analogues to the dishes and the cells were incubated for 3 or 24 h. The cells were then washed with PBS and collected. The cells were suspended with PBS and centrifuged, and the supernatant was removed. The cells were resuspended with PBS and clarified using 40 μm filters to prepare single cell suspensions. The single cells were analyzed by a Gallios flow cytometer. The experiment was performed three times.

### 2.6. Animal Experiments

#### 2.6.1. Animal and Tumor Model

The experimental protocols were approved by the Hokkaido University Animal Care Committee (15-0119, 5 October 2015) in accordance with the guidelines for the care and use of laboratory animals. BALB/c Slc-nu/nu nude mice (females) and ddY mice (females) were purchased from Japan SLC, Inc. (Hamamatsu, Japan).

We injected 5–10 million BxPc3 cells in PBS subcutaneously in the right or left shoulder. The mice were used for in vivo experiments after the tumor size reached 100 mm^3^. During procedures, mice were anesthetized with isoflurane.

#### 2.6.2. In Vivo and Ex Vivo Fluorescence Imaging

The tumor-bearing mice were given an intravenous injection of 0.1 mL of 100 μM Cy5/sulfoCy5-peptide A. Ten minutes after the injection, the fluorescence images were taken from a side view by FluorVivo. After imaging, the mice were euthanized, and the organs were collected and imaged by FluorVivo. The obtained images were analyzed using ImageJ software ver. 1.51. The experiment was performed three times.

#### 2.6.3. Measurement of the Concentration of Dye-Peptide A in Blood

The nude mice were administered an intravenous injection of 0.1 mL of 100 μM Cy5/sulfoCy5-peptide A. Subsequently, blood was collected from the tail vein with a heparinized microhematocrit capillary tube (Terumo, Tokyo, Japan) at 5, 10, 15, 20, 30, 40, and 60 min. Then, the tube was sealed and the plasma was separated. The fluorescence intensity of Cy5 dyes in the plasma was observed using fluorescence microscopy. The obtained images were analyzed using ImageJ software ver. 1.51. The experiment was performed three times.

#### 2.6.4. Stability of Dye-Peptide A in Mouse Plasma

Blood was collected from ddY mice and mixed well with the solution containing heparin (approximately 1.2 U/mL). The mixture was centrifuged for 20 min at 4 °C and the supernatant was collected as mouse plasma, which was preserved at 4 °C until use. We dissolved 1 μM (final) Cy5/sulfoCy5-peptide A in the mouse plasma and the plasma was incubated for 1 or 10 min at 37 °C. Subsequently, MeOH was added to the plasma and mixed well. Then, the mixture was put in a cool, dark place for 15 min and centrifuged for another 15 min at 37 °C at 1700× *g*. The obtained supernatant was analyzed with HPLC (A:B = 70:30 to 0:100 in 20 min for Cy5-peptide A, A:B = 80:20 to 20:80 in 25 min for sulfoCy5-peptide A). 

## 3. Results

### 3.1. Synthesis of Fluorescently Labeled Peptide A

**Figure 1:**
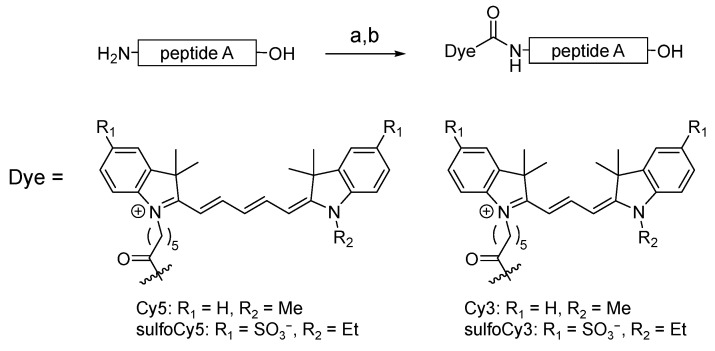
Synthetic scheme of dye-peptide A conjugates. Conditions and reagents: (**a**) Dye-SE, DMSO, 0.1 M Na_2_HPO_4_aq.; (**b**) TFA.

Cy3/Cy5-peptide A and sulfoCy3/sulfoCy5-peptide A were synthesized using commercially available Cy3/Cy5 succinimidyl ester (SE) and sulfoCy3/sulfoCy5 SE (Figure 1). These dyes were conjugated with peptide A through an amide bond at their N-termini. The dye-peptide A conjugates were purified by HPLC and characterized by MALDI-TOF-MS. Dye-SE and dye-peptide A were analyzed by HPLC under the same conditions; the retention times are shown in Table 1 because they are indicators of hydrophilicity/lipophilicity. The data suggested that the hydrophilicity and lipophilicity of Cy3/Cy5 and sulfoCy3/sulfoCy5 are quite different.

### 3.2. In Vitro Analysis of Dye-Peptide A by Microscopy and Flow Cytometry

**Figure 2:**
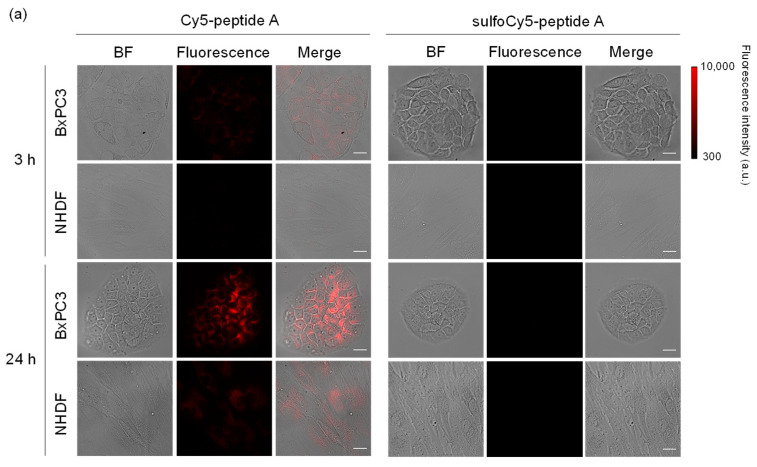

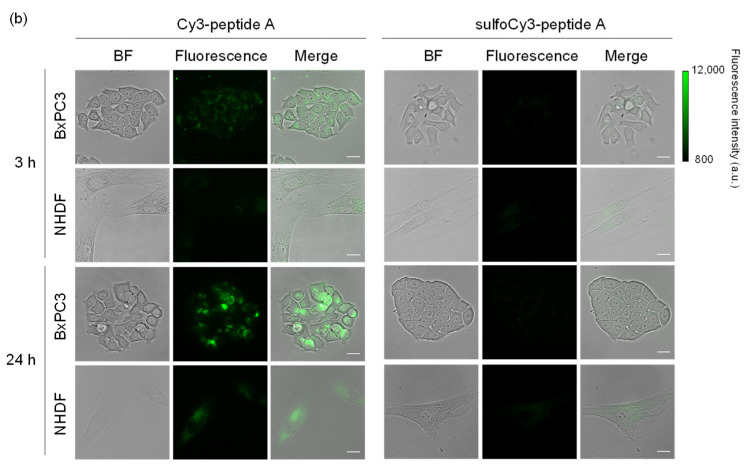
Fluorescence images of BxPC3 and NHDF cells treated with dye-peptide A. (**a**) Cy5/sulfoCy5-peptide A, (**b**) Cy3/sulfoCy3-peptide A. Cells were incubated with 0.1 μM dye-peptide A at 37 °C for 3 or 24 h before imaging. Bright field (BF), Cy5/Cy3 fluorescence, and merge images are shown. Scale bar: 10 μm.

**Figure 3:**
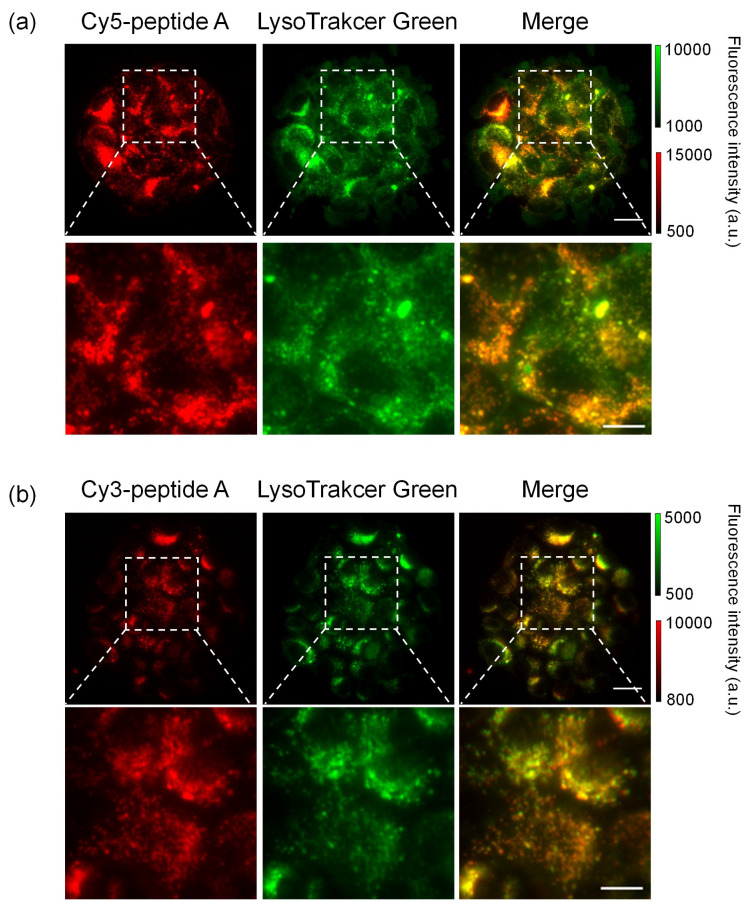
Colocalization of dye-peptide A and LysoTracker Green. (**a**) Cy5-peptide A, (**b**) Cy3-peptide A. Scale bar: 10 μm, 5 μm for magnified images.

**Figure 4:**
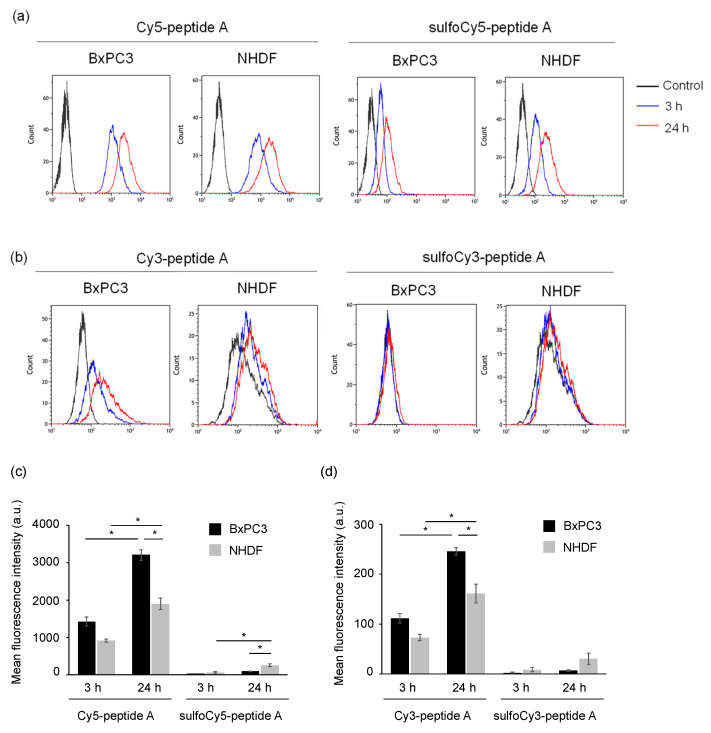
Flow cytometry of BxPC3 and NHDF cells treated with dye-peptide A. The histograms of (**a**) Cy5/sulfoCy5-peptide A and (**b**) Cy3/sulfoCy3-peptide A. The analysis of (**c**) Cy5/sulfoCy5-peptide A and (**d**) Cy3/sulfoCy3-peptide A. Cells were incubated with 0.1 μM dye-peptide A at 37 °C for 3 or 24 h before flow cytometry. The data represent the mean ± SEM (*n* = 3). The statistical significance was assessed by Tukey-Kramer test (* *p* < 0.01).

The uptake of dye-peptide A conjugates into BxPC3 cells was investigated in vitro using fluorescence microscopy and flow cytometry (FCM). As a result of microscopy, after incubation of Cy3/Cy5-peptide A, a fluorescent signal was observed inside the BxPC3 cells; however, there was much less uptake of sulfoCy3/sulfoCy5-peptide A, suggesting that the negative charge and hydrophilicity inhibited the cell uptake of the peptides (Figure 2a,b). The fluorescence intensities of the conjugates in cells increased with time, showing that the dye-peptide A gradually accumulated in cells. The fluorescent intensity in BxPC3 cells at 24 h was higher than that in the control cells, NHDF cells, suggesting the cell specificity of peptide A towards pancreatic cancer (Figure 2). The sub-cellular localization of Cy3/Cy5-peptide A in BxPC3 cells appeared to be lysosomal. The localization was examined with a commercial lysosome marker, LysoTracker Green, and showed that the fluorescence signals of Cy3/Cy5-peptide A and the marker overlapped well (Figure 3a,b). In FCM, as seen in fluorescence microscopy, the accumulation of C3/Cy5-peptide A in BxPC3 cells was much greater than the accumulation of sulfoCy3/sulfoCy5-peptide A (Figure 4a–d). Additionally, a quantitative analysis of FCM showed that the accumulation of Cy3/Cy5-peptide A conjugates in BxPC3 cells at 24 h was significantly higher than that in BxPC3 cells at 3 h and that in NHDF cells at 24 h. These results indicated that the dye-peptide A specifically accumulated in BxPC3 cells over time and the accumulation was influenced by the chemical properties of the fluorescent dyes, such as charge and hydrophilicity/lipophilicity.

### 3.3. In Vivo and Ex Vivo Analysis of Dye-Peptide A Conjugates

**Figure 5:**
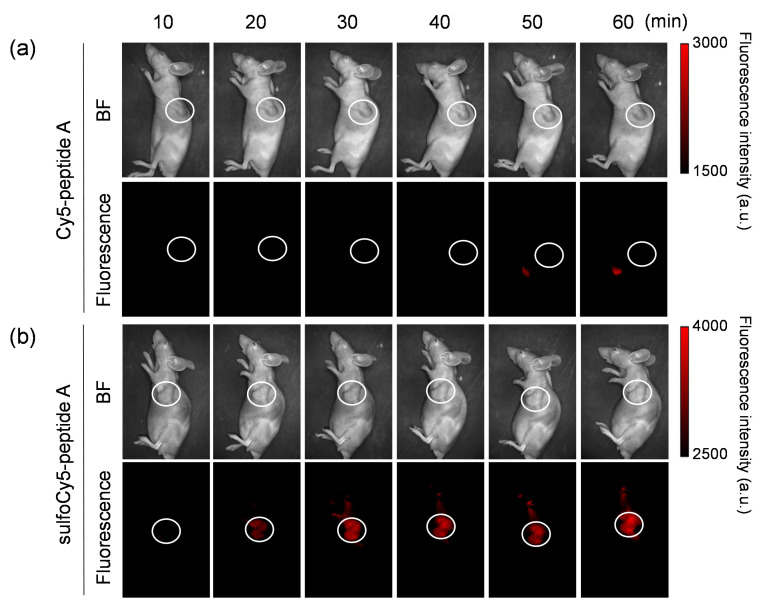
In vivo fluorescence imaging of tumor-bearing mice injected with dye-peptide A. The tumor-bearing mice were intravenously given 0.1 mL of (**a**) 100 μM Cy5-peptide A and (**b**) 100 μM sulfoCy5-peptide A. Ten minutes after the injection, the images were taken from a side view. The tumor is indicated by white circles. The in vivo experiment was performed three times.

**Figure 6:**
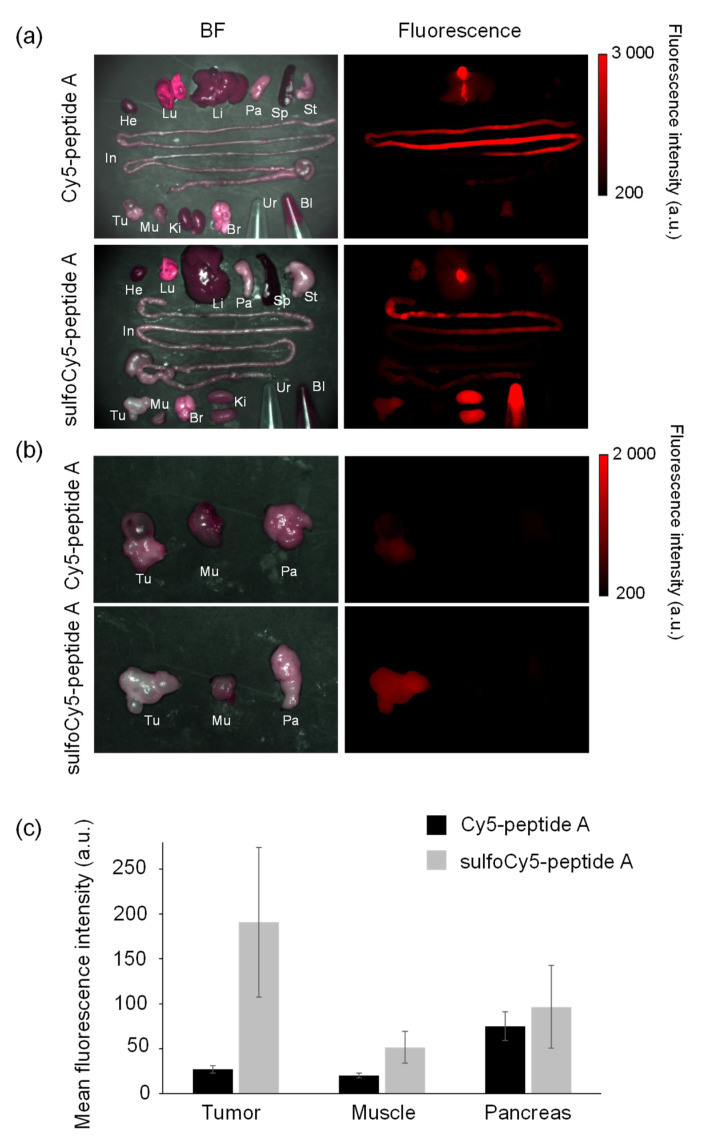
Ex vivo fluorescence imaging of organs collected from tumor-bearing mice injected with dye-peptide A. He, heart; Lu, lung; Li, liver; Pa, pancreas; Sp, spleen; St, stomach; In, intestine; Br, brain; Ki, kidney; Mu, muscle; Tu, tumor; Ur, urine; Bl, blood. Comparison of (**a**) all organs, urine, and blood, and (**b**) tumors, muscle, and pancreases. (**c**) The comparison of the mean fluorescence intensity of tumors, muscle and pancreases. The data represent the mean ± SEM (*n* = 3).

**Figure 7:**
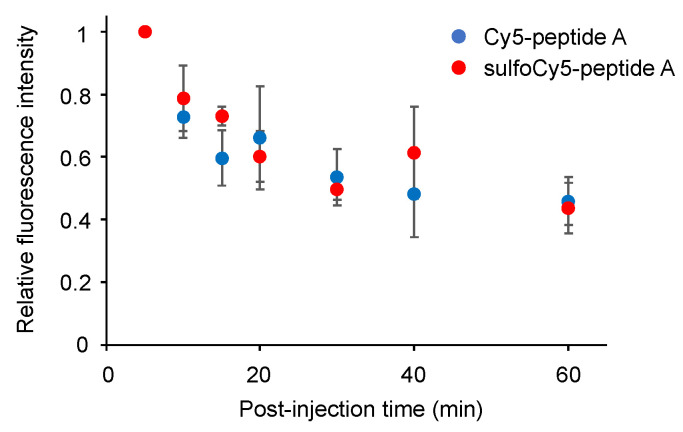
The time course of the concentration of Cy5/sulfoCy5-peptide A in mouse plasma. The data represent the mean ± SEM (*n* = 3).

The biodistribution and in vivo kinetics of Cy5/sulfoCy5-peptide A in animals were examined. The in vivo fluorescence images of tumor-bearing mice injected by Cy5/sulfoCy5-peptide A are shown in Figure 5a,b. After injection of Cy5-peptide A via the tail vein, almost no fluorescence was detected in the tumor, even at 60 min. Fluorescence was observed in the abdomen at 50–60 min. Conversely, in tumor-bearing mice injected with sulfoCy5-peptide A, the time-dependent fluorescent signal was observed in the tumors, although there was a non-specific fluorescence signal from the body’s surface. The ex vivo fluorescence imaging of various organs, urine, and blood from mice injected with Cy5-peptide A showed intense fluorescent signals in the intestines, livers, and kidneys, suggesting that the fluorescence from the abdomen in in vivo imaging was derived from these organs, mainly the intestines (Figure 6a). In accordance with in vivo imaging, the accumulation in tumor tissue was not observed. In the case of sulfoCy5-peptide A, strong fluorescence was observed in the intestines, livers, and kidneys, as with Cy5-peptide A, and fluorescence was detected from tumor tissue and the lungs, in contrast to Cy5-peptide A (Figure 6b). When tumors, muscle, and pancreases from mice injected with Cy5/sulfoCy5-peptide A were compared, a strong fluorescence intensity was observed from tumor tissue collected from mice injected with sulfoCy5-peptide A (Figure 6c). There was much less fluorescence in the muscle and pancreases from mice injected with both Cy5/sulfoCy5-peptide A, suggesting that the dye-peptide A did not accumulate in normal cells. When the concentration of Cy5/sulfoCy5-peptide A in blood was examined after injection, the change over time was almost the same (Figure 7). These results show that sulfoCy5-peptide A accumulated in tumors but Cy5-peptide A did not, which was not consistent with the in vitro experiment.

### 3.4. Stability of Dye-Peptide A Conjugates in Plasma

**Figure 8:**
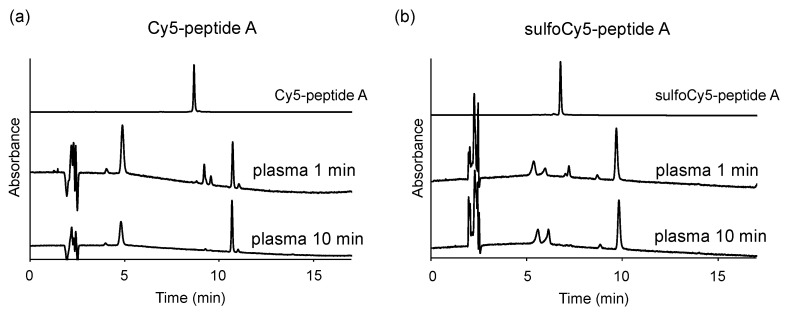
Stability of dye-peptide A in mice plasma. (**a**) 1 μM Cy5-peptide A and (**b**) 1 μM sulfoCy5-peptide A were dissolved in the plasma. The plasma containing dye-peptide A was incubated at 37 °C for 1 min or 10 min.

To investigate the difference in uptake of dye-peptide A between in vitro and in vivo, the stability of the peptides in the plasma was examined. They were incubated in mice plasma at 37 °C and analyzed with HPLC at a wavelength of 649 nm, which is the maximum absorption of Cy5/sulfoCy5. As a result, both conjugates were digested within 1 min, and the digestion ended in 10 min, producing some degraded compounds (Figure 8a,b).

## 4. Discussion

In fluorescence imaging, the targeting efficiency of dye-ligand conjugates is affected by the chemical properties of the fluorescent dyes. So far, the effect of the chemical properties of the dye on in vitro and in vivo cell uptake has mainly been investigated using arginylglycylaspartic acid (RGD) peptide, which binds to integrin α_v_β_3_ [11,13,16], and whose expression is associated with cancer malignancy [17,18,19]. However, other peptides have not been systematically investigated. In this study, we used a CPP that penetrates the cell membrane and is taken into the cells, not a binding peptide such as RGD. We investigated whether in vitro and in vivo cell uptake with dye-CPP conjugates changed when Cy3/Cy5 and negatively charged sulfoCy3/sulfoCy5 dyes were used as the fluorescent dyes. 

With these dye-peptide A conjugates, fluorescence microscopy and FCM were performed as in vitro experiments to investigate cell uptake. As a result of these experiments, a time-dependent accumulation of Cy3/Cy5-peptide A in BxPC3 cells was observed, although there was almost no accumulation of sulfoCy3/sulfoCy5-peptide A (Figure 2 and Figure 4). Additionally, the time course of the cell uptake was almost the same when Cy3-peptide A and Cy5-peptide A were added to BxPC3 cells (Figure 4). These results showed that the in vitro cell uptake of peptide A was strongly influenced by the charge and hydrophilicity/lipophilicity of the fluorescent dyes, as the main difference between Cy3/Cy5 and sulfoCy3/sulfoCy5 is the presence or absence of sulfonate groups on their heterocyclic rings. Additionally, the introduction of the sulfonate groups to the heterocyclic ring of the cyanine dye increased hydrophilicity, leading to a lower distribution of Cy3/Cy5 fluorescence cores in the lipophilic cell membrane. Therefore, sulfoCy3/sulfoCy5-peptide A are unlikely to be close to the cell membrane and exhibit lower cell-penetrating properties. 

In fluorescence microscopy, Cy5-peptide A formed dot-like structures in BxPC3 cells. The colocalization with LysoTracker Green revealed that Cy5-peptide A was localized in lysosome, indicating that the main mechanism of the cell uptake is endocytosis (Figure 3) [20]. However, the target molecules involved in endocytosis were unclear. Further analysis is needed to elucidate the mechanism.

To investigate the biodistribution and in vivo kinetics of Cy5/sulfoCy5-peptide A conjugates, we performed in vivo fluorescence imaging, ex vivo imaging of various organs, and measurement of the dye concentration in blood (Figure 5, Figure 6 and Figure 7). The fluorescence intensity in in vivo fluorescence imaging is affected by the depth of the tumors; however, in this study, cancer cells were implanted by subcutaneous injection and the tumor size was not large. Therefore, it was considered that there was little influence of the depth of the tumors. The fluorescence imaging showed that sulfoCy5-peptide A accumulated in tumor tissue, but Cy5-peptide A did not. Additionally, Cy5-peptide A was eliminated via hepatic clearance more than renal clearance, whereas sulfoCy5-peptide A exhibited the opposite pattern. A stability analysis of Cy5/sulfoCy5-peptide A in mice plasma revealed that Cy5/sulfoCy5-peptide A were rapidly digested (Figure 8). The results of the stability analysis suggested that the in vivo fluorescence images mainly represented the degradation products due to the rapid degradation in plasma. Furthermore, Cy5-peptide A and sulfoCy5-peptide A degraded differently in plasma (Figure 8). Therefore, the charge and hydrophilicity/lipophilicity of the fluorescent dyes would also affect the degradation of the conjugates by enzymes in the plasma. The reason that the digested sulfoCy5-peptide A accumulated in tumor tissue is unknown; it is possible that the digested products still possess the binding property in BxPC3 cells, and that the products are more accessible to the surface of cancer cells because they can decrease non-specific interactions to the proteins in blood due to negatively-charged and hydrophilic properties [11]. It was investigated whether fluorescent dyes affected the target efficiency and pharmacokinetics using conjugates with the RGD sequence as a ligand [11,13,16]. In the report on biodistribution of imaging regents using cyclic RGD as a carrier molecule and a variety of cyanine dyes as labeling molecules, imaging regents with sulfonated cyanine dyes showed greater accumulation in tumor tissue and less hepatic elimination than those with non-sulfonated cyanine dyes [13]. It appears that the presence of a sulfonate group would have a better effect on accumulation in tumor tissue. 

Our results showed that the charge and hydrophilicity/lipophilicity of fluorescent dyes can influence in vitro and in vivo cell uptake in different ways, although the in vitro and in vivo cell uptakes with dye-RGD are consistent with previous reports [11]. There are some cases in which positively charged or lipophilic fluorescent dyes may lead to poor in vivo targeting efficiency of peptides, despite their favorable properties for in vitro cell uptake. Other factors such as flexibility, charge balance, polar surface area, and the molecular size of the entire compound can affect the cell uptake. These factors can be investigated by calculation. Accordingly, attention should be paid to the chemical properties of fluorescent dyes used in fluorescence imaging based on a targeting strategy.

## 5. Conclusions

Herein, we investigated the influence of fluorescent dyes on in vitro and in vivo cell uptake of dye-CPP conjugates. We used peptide A as a model peptide and Cy3/Cy5 and sulfoCy3/sulfoCy5 as the fluorescent dyes. Our results showed that the charge and hydrophilicity/lipophilicity of fluorescent dyes affected the targeting efficiency. Positive charge and lipophilicity were advantageous to in vitro cell uptake, but the opposite result was obtained in vivo. Accordingly, although it is not always true in the case of different peptides, dyes, and cell lines, it is important to consider the chemical properties of fluorescent dyes when designing fluorescent imaging reagents with ligands.

## Figures and Tables

**Table 1:** Retention times of dye-SE and dye-peptide A conjugates.

Compound	Retention Time (min) ^1^
Cy5-SE	22.6
sulfoCy5-SE	9.3
Cy3-SE	20.8
sulfoCy3-SE	7.3
Cy5-peptide A	14.1
sulfoCy5-peptide A	6.8
Cy3-peptide A	12.8
sulfoCy3-peptide A	4.4

^1^ Obtained from HPLC charts in Figure S1.

## Data Availability

The data presented in this study are available on request from the corresponding author.

## Supplementary Materials

The following are available online at https://www.mdpi.com/2072-6694/13/9/2245/s1, Figure S1: HPLC charts of dye-SE and dye-peptide A conjugates.
