# Correction: Takakura et al. In Vitro and In Vivo Cell Uptake of a Cell-Penetrating Peptide Conjugated with Fluorescent Dyes Having Different Chemical Properties. *Cancers* 2021, *13*, 2245

**DOI:** 10.3390/cancers14081880

**Published:** 2022-04-08

**Authors:** Hideo Takakura, Honoka Sato, Kohei Nakajima, Motofumi Suzuki, Mikako Ogawa

**Affiliations:** Laboratory of Bioanalysis and Molecular Imaging, Graduate School of Pharmaceutical Sciences, Hokkaido University, Sapporo 060-0812, Hokkaido, Japan; htakakura@pharm.hokudai.ac.jp (H.T.); h_sato_2134@frontier.hokudai.ac.jp (H.S.); knakajima@pharm.hokudai.ac.jp (K.N.); suzukimo@hirakata.kmu.ac.jp (M.S.)

There was an error in the description in the original publication, and it contained information that should not have been disclosed [1].

Reference [9] does not contain information on the peptide. The inventors of the peptide have not disclosed the peptide sequence in any scientific papers yet. We obtained the information only through private communication and published it without their agreement.

Therefore, all the concrete information about the peptide, including its sequence, should have been removed from the original publication. The structure of the peptide has been concealed and the name replaced by Peptide A. The research work is scientifically valid and unchanged. No corrections regarding the testing of the peptide were made. 

Thus, we raised the correction as below:

1. Replace all peptide designation with “peptide A” in the main text. The relevant information for it also needs to be updated.
2. Delete “using mRNA display technology for BxPC3 cells, human pancreatic ductal adenocarcinoma [9].” in the last paragraph of the Introduction.
3. Section 2.2 should be corrected as below:

Cy5-peptide A. Peptide A (0.74 mg) and Cy5 succinimidyl ester (SE) (0.20 mg, 0.32 μmol) were dissolved in 0.1 mL of DMSO, and 0.1 mL of 1 mM Na_2_HPO_4_aq was added to the solution. The reaction mixture was stirred for 1 h at 50 °C. Then, 0.2 mL of TFA was added to the reaction mixture. The mixture was further stirred for 30 min at 50 °C. The product was purified by semi-preparative reverse-phase HPLC (eluent A: H_2_O/1% TFA, eluent B: 99% CH_3_CN/0.1% TFA, A:B = 70:30 to 0:100 in 20 min) to produce a blue solid (0.41 mg). The HPLC chart of Cy5-peptide A is shown in Figure S1.

sulfoCy5-peptide A. sulfoCy5-peptide A was synthesized from peptide A and sulfoCy5-SE by the same method as that used to obtain Cy5-peptide A. The product was purified by semi-preparative reverse-phase HPLC (A:B = 80:20 to 20:80 in 25 min) to produce a blue solid. The HPLC chart of sulfoCy5-peptide A is shown in Figure S1.

Cy3-peptide A. Cy3-peptide A was synthesized from peptide A and Cy3-SE by the same method as that used to obtain Cy5-peptide A. The product was purified by semi-preparative reverse-phase HPLC (A:B = 70:30 to 0:100 in 20 min) to produce a red solid. The HPLC chart of Cy3-peptide A is shown in Figure S1.

sulfoCy3-peptide A. sulfoCy3-peptide A was synthesized from peptide A and sulfoCy3-SE by the same method as that used to obtain Cy5-peptide A. The product was purified by semi-preparative reverse-phase HPLC (A:B = 80:20 to 20:80 in 25 min) to produce a red solid. The HPLC chart of sulfoCy3-peptide A is shown in Figure S1.

4. Figure 1 should be corrected as below:

5. Figure 2 should be updated as below:

6. Figure 3 should be updated as:

7. Figure 4 should be updated as:

8. Figure 5 should be updated as:

9. Figure 6 is updated as:

10. Figure 7 is updated as:

11. Figure 8 should be updated as:

12. Change the supplementary information

Figure S1 should be:

**Figure S1:**
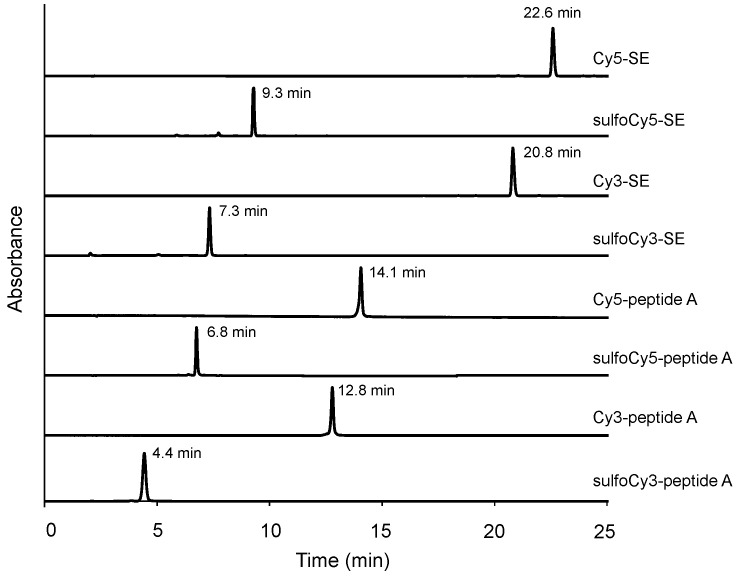
HPLC charts of dye-SE and dye-peptide A conjugates.

Figure S2 should be removed. Since some references have been deleted, the citation of them should be removed from the main text accordingly.

The authors apologize for any inconvenience caused and state that the scientific conclusions are unaffected. The original publication has also been updated.

## Figures and Tables

**Figure 1:**
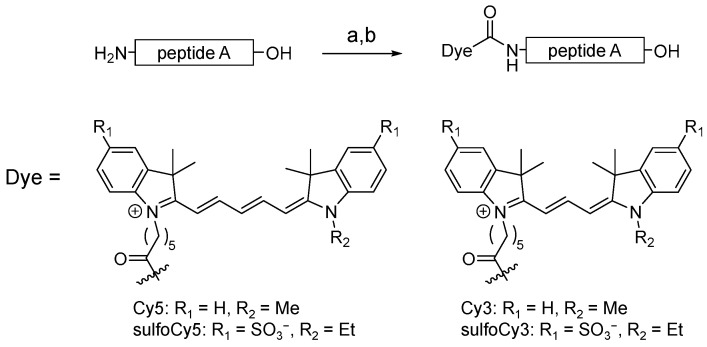
Synthetic scheme of dye-peptide A conjugates. Conditions and reagents: (a) Dye-SE, DMSO, 0.1 M Na_2_HPO_4_aq.; (b) TFA.

**Figure 2:**
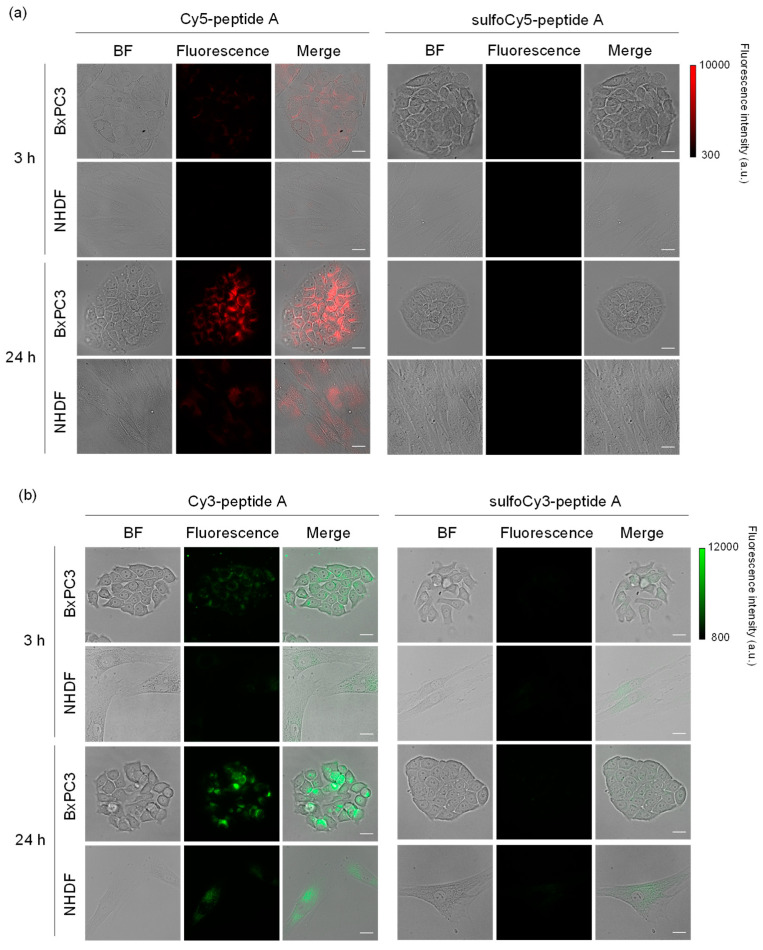
Fluorescence images of BxPC3 and NHDF cells treated with dye-peptide A. (**a**) Cy5/sulfoCy5-peptide A, (**b**) Cy3/sulfoCy3-peptide A. Cells were incubated with 0.1 μM dye-peptide A at 37 °C for 3 or 24 h before imaging. Bright field (BF), Cy5/Cy3 fluorescence, and merge images are shown. Scale bar: 10 μm.

**Figure 3:**
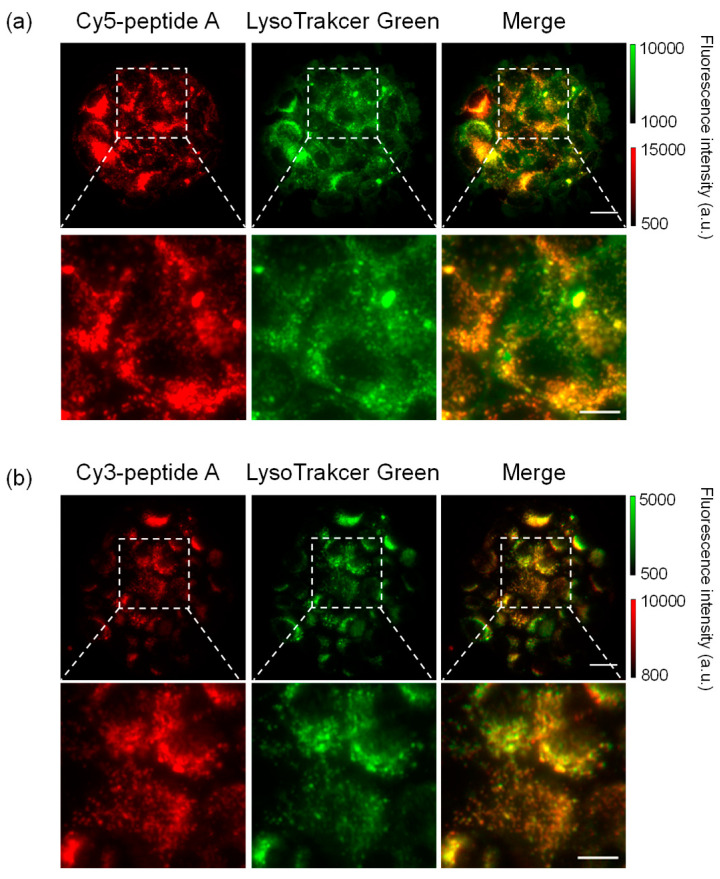
Colocalization of dye-peptide A and LysoTracker Green. (**a**) Cy5-peptide A, (**b**) Cy3-peptide A. Scale bar: 10 μm, 5 μm for magnified images.

**Figure 4:**
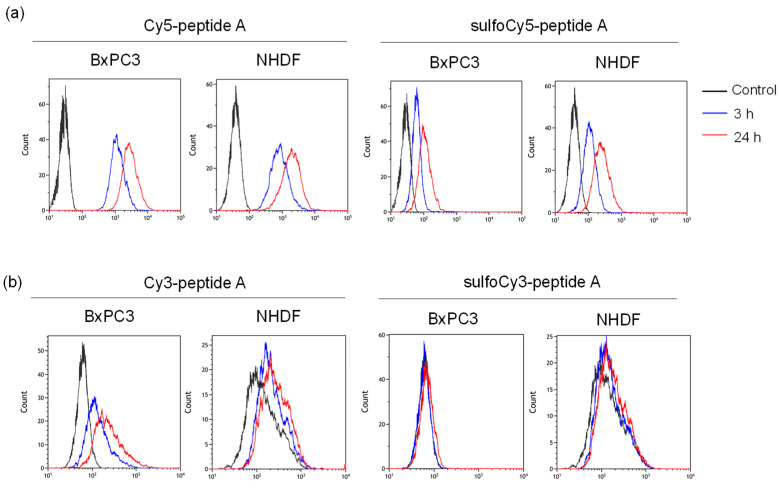

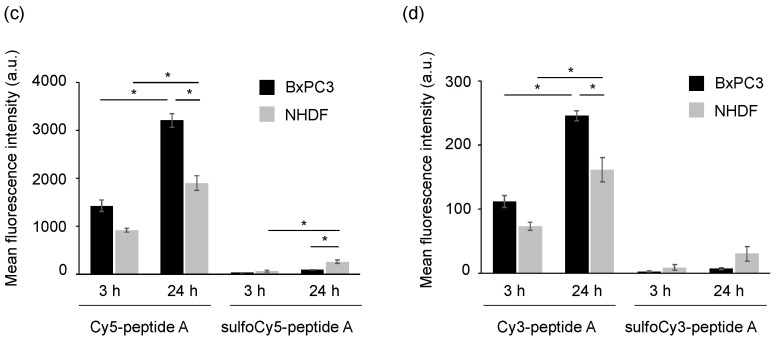
Flow cytometry of BxPC3 and NHDF cells treated with dye-peptide A. The histograms of (**a**) Cy5/sulfoCy5-peptide A and (**b**) Cy3/sulfoCy3-peptide A. The analysis of (**c**) Cy5/sulfoCy5-peptide A and (**d**) Cy3/sulfoCy3-peptide A. Cells were incubated with 0.1 μM dye-peptide A at 37 °C for 3 or 24 h before flow cytometry. The data represent the mean ± SEM (*n* = 3). The statistical significance was assessed by Tukey-Kramer test (* *p* < 0.01).

**Figure 5:**
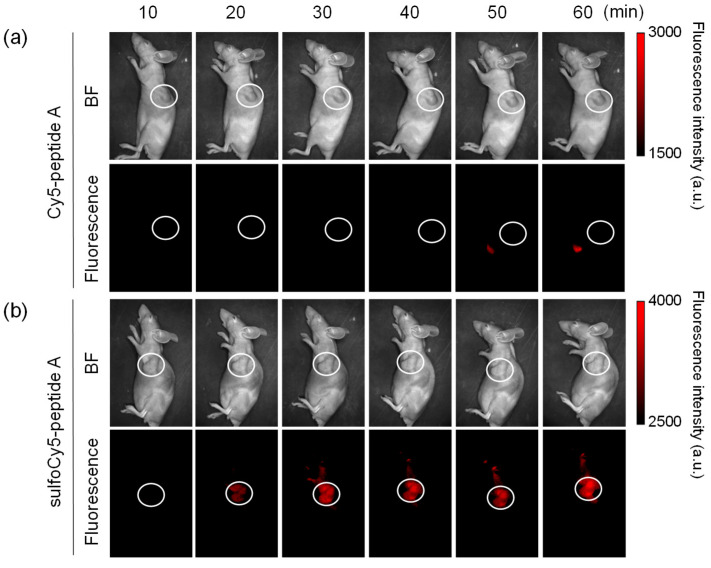
In vivo fluorescence imaging of tumor-bearing mice injected with dye-peptide A. The tumor-bearing mice were intravenously given 0.1 mL of (**a**) 100 μM Cy5-peptide A and (**b**) 100 μM sulfoCy5-peptide A. Ten minutes after the injection, the images were taken from a side view. The tumor is indicated by white circles. The in vivo experiment was performed three times.

**Figure 6:**
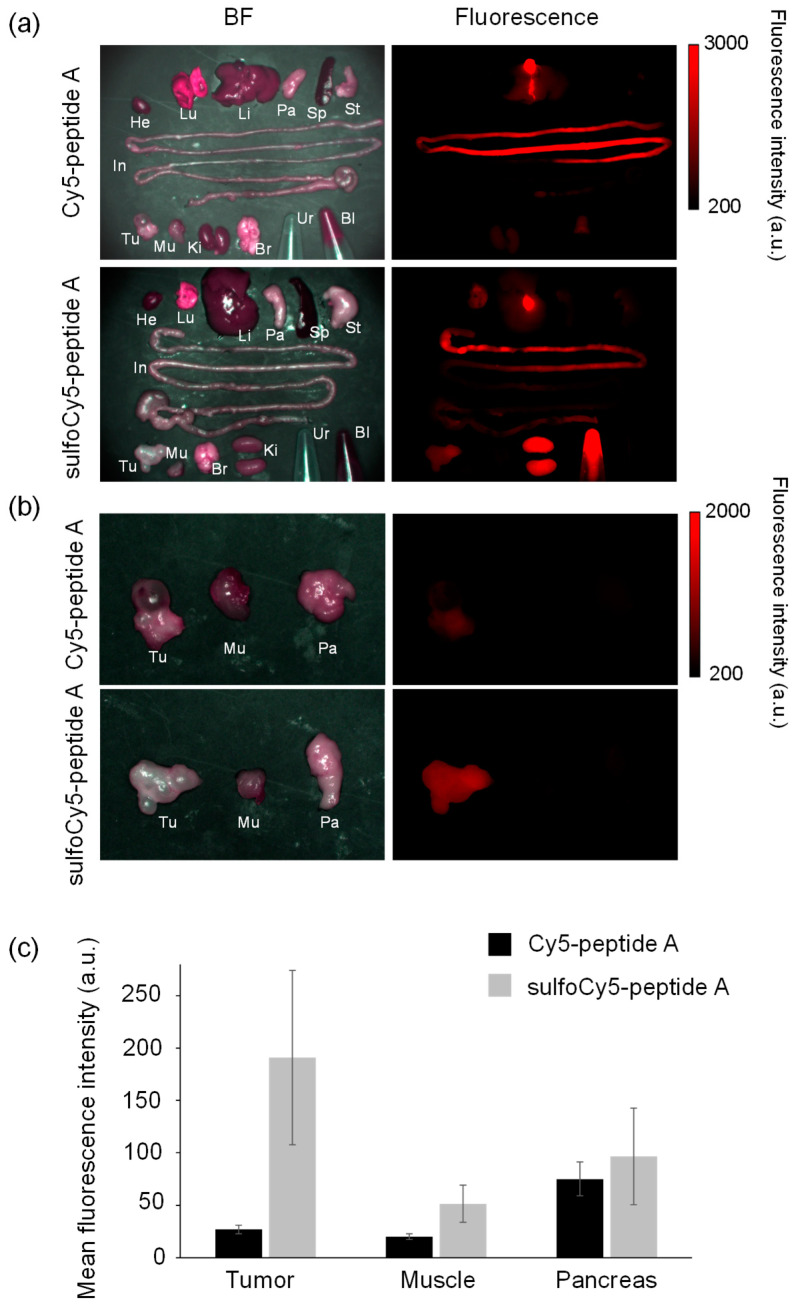
Ex vivo fluorescence imaging of organs collected from tumor-bearing mice injected with dye-peptide A. He, heart; Lu, lung; Li, liver; Pa, pancreas; Sp, spleen; St, stomach; In, intestine; Br, brain; Ki, kidney; Mu, muscle; Tu, tumor; Ur, urine; Bl, blood. Comparison of (**a**) all organs, urine, and blood, and (**b**) tumors, muscle, and pancreases. (**c**) The comparison of the mean fluorescence intensity of tumors, muscle, and pancreases. The data represent the mean ± SEM (*n* = 3).

**Figure 7:**
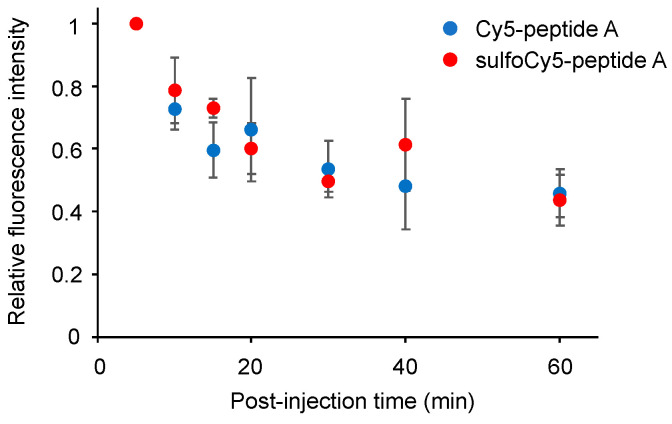
The time course of the concentration of Cy5/sulfoCy5-peptide A in mouse plasma. The data represent the mean ± SEM (*n* = 3).

**Figure 8:**
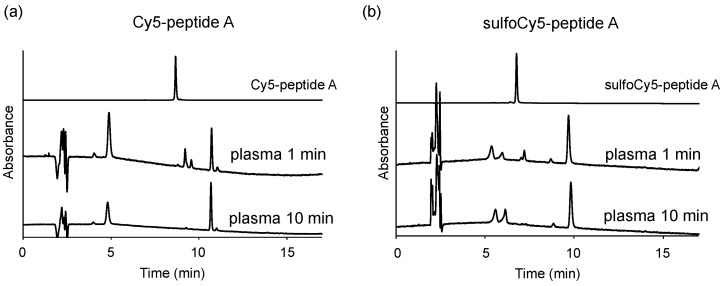
Stability of dye-peptide A in mice plasma. (**a**) 1 μM Cy5-peptide A and (**b**) 1 μM sulfoCy5-peptide A were dissolved in the plasma. The plasma containing dye-peptide A was incubated at 37 °C for 1 min or 10 min.
