# Supplementary material for: In Vitro and In Vivo Cell Uptake of a Cell-Penetrating Peptide Conjugated with Fluorescent Dyes Having Different Chemical Properties
Source: Cancers (Basel). 2021 May 7;13(9):2245. doi: 10.3390/cancers13092245 (PMC8124942; doi:10.3390/cancers13092245)
Supplement: Supplementary file 1 [file cancers-13-02245-s001.zip › cancers-1194699-final-suppl.pdf]

Supplementary Materials: In Vitro and In Vivo Cell Uptake of a Cell-Penetrating Peptide Conjugated with Fluorescent Dyes Having Different Chemical Properties

Hideo Takakura, Honoka Sato, Kohei Nakajima, Motofumi Suzuki and Mikako Ogawa

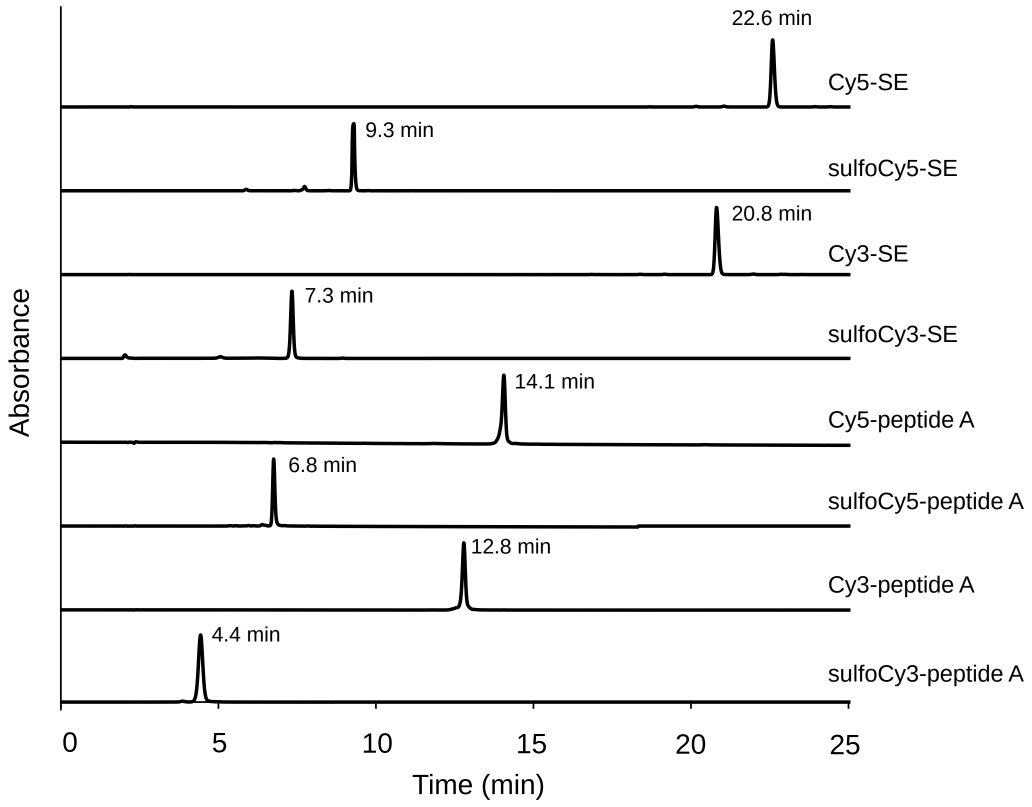

Figure S1. HPLC charts of dye-SE and dye-peptide A conjugates. The compounds were analyzed by reverse-phase HPLC (eluent A: H_2O/0.1% trifluoro acetic acid (TFA), eluent B: CH_3CN/0.1%TFA, A:B = 80:20 to 20:80 in 25 min). The detection wavelengths were 649 nm and 545 nm for Cy5/sulfoCy5 and Cy3/sulfoCy3, respectively. The retention time is indicated. .
